# Real-Time Monitoring of miRNA Function in Pancreatic Cell Lines Using Recombinant AAV-Based miRNA Asensors

**DOI:** 10.1371/journal.pone.0066315

**Published:** 2013-06-11

**Authors:** Jing Chen, Xinjuan Liu, Xue Chen, Zihao Guo, Juan Liu, Jianyu Hao, Jie Zhang

**Affiliations:** 1 Department of Gastroenterology, Beijing Chaoyang Hospital, Capital Medical University, Chaoyang District, Beijing, China; 2 Management of Hospital Infection, Zhengzhou Tenth People's Hospital, Zhengzhou, Henan, China; National Institute for Viral Disease Control and Prevention, CDC, China

## Abstract

**Background:**

MicroRNAs (miRNAs) are reportedly involved in pancreatic ductal adenocarcinoma (PDAC) development. Current methods do not allow us to reliably monitor miRNA function. Asensors are adeno-associated virus (AAV) vector miRNA sensors for real-time consecutive functional monitoring of miRNA profiling in living cells.

**Methods:**

miR-200a, -200b, -21, -96, -146a, -10a, -155, and -221 in three PDAC cell lines (BxPC-3, CFPAC-1, SW1990), pancreatic epithelioid carcinoma cells (PANC-1), and human pancreatic nestin-expressing cells (hTERT-HPNE) were monitored by Asensors. Subsequently, the real-time consecutive functional profile of all miRNAs was evaluated.

**Results:**

Selected miRNAs were detectable in all cell lines with high sensitivity and reproducibility. In the three PDAC cell lines, BxPC-3, CFPAC-1, and SW1990, the calibrated signal unit of the eight miRNAs Asensors was significantly lower than that of the Asensor control. However, in PANC-1 cells, miR-200a and -155 showed upregulation of target gene expression at 24 hours after infection with the sensors; at 48 hours, miR-200b and -155 displayed upregulation of reporter expression; and at 72 hours, reporter gene expression was upregulated by miR-200a and -200b. The result that miRNA could upregulate gene expression was further confirmed in miR-155 of hTERT-HPNE cells. Furthermore, miRNA activity varied among cell/tissue types and time.

**Conclusion:**

It is possible that miRNA participates in the pathophysiology of pancreatic cancer, but the current popular methods do not accurately reveal the real-time miRNA function. Thus, this report provided a convenient, accurate, and sensitive approach to miRNA research.

## Introduction

Pancreatic ductal adenocarcinoma (PDAC) is a highly malignant cancer with increasing incidence and mortality worldwide. It is one of the major leading causes of cancer-related mortality with a five-year survival rate of 6–7% [1]. Because of its insidious onset, only 7% of cases present in the early stages of disease, and the late diagnosis leads to a low resection rate and poor prognosis [2]. Therefore, further research on the pathophysiology of PDAC is a top priority for PDAC control and prevention.

MicroRNAs (miRNAs) are noncoding RNAs that are 18–25 nucleotides long. Recently, they have emerged as a critical class of negative regulators of gene expression through the modulation of post-transcriptional activity of multiple target mRNAs. They regulate gene expression via complementarity with the 3′-untranslated region (3′-UTR) of their target mRNAs. miRNAs regulate gene expression either by target mRNA degradation, repression of its translation, or sometimes by upregulation of the target gene. More than 50% of the known miRNAs have been shown to participate in human tumorigenesis and/or metastasis by directly targeting oncogenes or tumor suppressor genes [3], [4]. Therefore, research focused on the role of miRNA in PDAC is rapidly increasing.

A number of methods, including Northern blot [5], real-time polymerase chain reaction (RT-PCR) [6], and microarrays [7] have been developed to detect and quantify miRNA expression, but they do not reflect the real-time and consecutive function of a given miRNA in living cells. In addition, miRNA expression levels do not always reflect the actual activity of each miRNA [8], [9]. The latter correlates with mature miRNA functions and is often affected by multiple steps along the miRNA pathway, including the miRNA-induced silencing complex (miRISC) forming efficiency, the binding affinity of miRNA to the target sequences at the 3′-UTR, and the inhibition efficiency through miRISC binding [10]. Therefore, if achievable, functional miRNA profiling, which reflects the real miRNA activity, may display many advantages over conventional miRNA profiling.

An “Asensor” is a recombinant adeno-associated virus (rAAV) vector miRNA sensor for real-time consecutive functional monitoring of miRNA profiling in living cells, constructed by inserting a given miRNA target sequence into the 3′-UTR of reporter genes and containing two independent expression cassettes encoding Gaussia luciferase (Gluc) and firefly luciferase (Fluc). Using the Asensors, miRNA activity can be inferred by measuring the inhibition of reporter gene expression. In this study, the real-time miRNA activity of miR-200a, -200b, -21, -96, -146a, -10a, -155, and -221 in three PDAC cell lines (BxPC-3, CFPAC-1, SW1990), one pancreatic epithelioid carcinoma (PANC-1), and human pancreatic nestin-expressing cells without causing cancer-associated changes (hTERT-HPNE) was monitored using their corresponding Asensors. Most previous research is consistent with the results reflected by the Asensors, yet Asensors provided new insights for some miRNAs, which may be important for worldwide miRNA research.

## Materials and Methods

### miRNA Asensor Construction

All Asensors were purchased from FivePlus Molecular Medicine Institute (Beijing, China). The miRNA Asensor array was established as previously reported [11]. The miRNA Asensor plasmid was constructed based on the AAV vector plasmid pAAV2neo and contained two independent expression cassettes encoding Fluc and Gluc [12]. The former was used to calibrate the transduction efficiency, while the latter, which included a miRNA perfect complementary target sequence in the 3′-UTR of Gluc, was used to monitor miRNA activity. A synthetic poly(A) signal/transcriptional pause site was inserted between the two expression cassettes and reduced the effects of spurious transcription on the Fluc reporter gene expression. miR-200a, -200b, -21, -96, -146a, -10a, -155, and -221 sensor plasmids were constructed by inserting one copy of the corresponding miRNA target sequence, miR-200a (UAACACUGUCUGGUAACGAUGU), miR-200b (UAAUACUGCCUGGUAAUGAUGA), miR-21 (UAGCUUAUCAGACUGAUGUUGA), miR-96 (UUUGGCACUAGCACAUUUUUGCU), miR-146a (UGAGAACUGAAUUCCAUGGGUU), miR-10a (UACCCUGUAGAUCCGAAUUUGUG), miR-155 (UUAAUGCUAAUCGUGAUAGGGGU), or miR-221 (AGCUACAUUGUCUGCUGGGUUUC), into the 3′-UTR of Gluc. They were then packaged into recombinant AAVs termed miRNA Asensors. The Asensor lacking the miRNA target sequence was used as a control. The Asensors were quantified by qPCR, and when the amount matched that of the Asensor-infected target cells, then the activity of miRNAs in cells paired with the target sequences of the Asensor was revealed by Gluc expediently, accurately, and sensitively.

### Cell Culture

Three PDAC cell lines (BxPC-3, CFPAC-1, SW1990), pancreatic epithelioid carcinoma (PANC-1), and human pancreatic nestin-expressing cells (hTERT-HPNE) were obtained from the American Type Culture Collection and grown under the recommended conditions, supplemented with 10% fetal bovine serum (Invitrogen, Carlsbad, CA) and 100 µM each of penicillin and streptomycin (Invitrogen, Carlsbad, CA) in a humidified atmosphere of 5% CO_2_ at 37°C.

### Monitoring of miRNAs

Preliminary experiments showed that 10,000 cells infected by 10^8^ copies of Asensors was the best ratio for monitoring miRNA activity in target cells and was applied in the following experiments. Cells were seeded in a 96-well cell culture plate (200 µl of recommended culture medium for each cell line) one day before infection. After infection with the Asensors, the target cells were further incubated for 3 days, and on each day, 20 µl of supernatant was sampled to detect Gluc, and 20 µl of culture medium was refilled to keep 200 µl of total culture medium. On day 3, cells were lysed to quantify the Fluc internal control.

### Assays of Fluc and Gluc Activity

The Gluc and Fluc assay kits were purchased from New England Biolabs (Ipswich, MA, USA) and Promega (Madison, WI, USA), respectively. Cells in the 96-well plates were spun down, and a 20 µl aliquot of the cell-free medium in each well was taken for Gluc activity assays at 24, 48, and 72 hours. Substrate solution (50 µl per well) of Gluc was added into the sample. For Fluc activity, 20 µl of cell lysate per well was added to the substrate solution (100 µl per well) of Fluc. Both Fluc and Gluc expression was then tested using a luminometer (Modulus™, Tuner BioSystems). The levels of Fluc and Gluc activity were quantified using relative light units (RLU).

### Calculation of Transduction Coefficients and Relative Inhibiting Folds

Although equal amounts of each miRNA Asensor were loaded into each well of the 96-well plate, the Fluc activity reflecting the transduction efficiency remained variable among different Asensors due to fluctuations in the titre for each Asensor. To solve this problem, the transduction coefficient (TC) was used to calibrate the miRNA activity obtained by each Asensor. The TC calculation for each Asensor was performed as follows. Without consideration of the miRNA repression of Gluc activity, the relationship between Fluc activity (F) and Gluc activity (G) could be approximated by
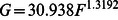
(1)


Then, the TC value, which is the ratio of G of Asensor_miRNA_ (G_miRNA_) to that of Asensor_control_ (G_control_) when no miRNA repression occurs, could be computed as follows: 
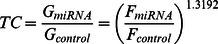
(2)


where G_control_ and G_miRNA_ represent the Gluc activity of the Asensor control and Asensor miRNA, respectively, and F_control_ and F_miRNA_ represent the Fluc activity of the Asensor control and Asensor miRNA, respectively.

The activity of miRNA in a cell line, represented by the relative inhibiting fold (RIF), was calculated by the formula
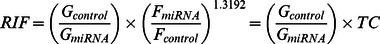
(3)


RIF represents the level of report gene expression regulated by miRNA compared with the control, which depends on the theory that “miRNAs inhibit target gene expression” [13].

### Statistical Analysis

Graphs were created with GraphPad Prism 6.0.1 software (San Diego, CA, USA). Differences in the RIF between miRNA and the control were tested for statistical significance by the independent-samples t test (SPSS version 17.0, Chicago, IL, USA). The level of significance was chosen as p<0.05.

## Results

### Assessment of Asensor Control

**Figure 1:**
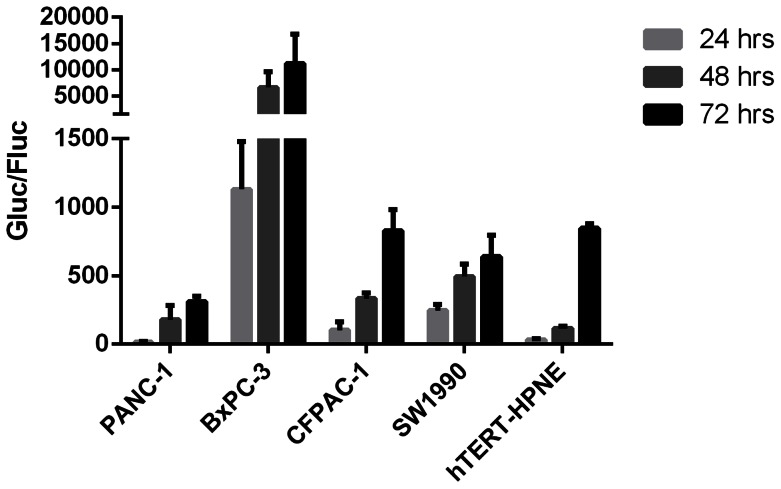
Assessment of Asensor control. Asensors without the target miRNA gene were used as controls to calibrate any system error or accidental error. The reporter luciferase was detected at 24, 48, and 72 hours after Asensor infection. miRNA activity was expressed as Gluc/Fluc. Control in BxPC-3 showed high level in all cell lines.

The Asensor was constructed as described in the Materials and Methods section. The Gluc expression Asensor without miRNA binding sites was also constructed as a control. To ensure that all rAAVs could act as an miRNA sensor, we first evaluated the Asensor control, and Gluc secreted into the supernatant was assayed at 24, 48, 72 hours after infection. As expected, we could detect high Gluc and Fluc signals in all cells. In this report, the infection efficiency of the Asensors, cell growth diversity, and other system and accidental errors were calibrated by Fluc, and miRNA activity was expressed as Gluc/Fluc. Although the Asensor control did not reflect any miRNA activity, to maintain consistency, the *y* axis in Figure 1 was also expressed as Gluc/Fluc. As shown in Figure 1, the Gluc/Fluc of the supernatants of BxPC-3 cells was consistently high, measuring 1130.51±350.93, 6633.16±2931.6, and 11143.97±5601.09 at 24, 48, and 72 hours, respectively. At 24, 48, and 72 hours, the Gluc/Fluc of PANC-1 was 14.85±5.59, 178.98±102.26, and 309.71±41.42, respectively; of hTERT-HPNE was 31.85±9.72, 115.53±14.20, and 845.77±31.66, respectively; of CFPAC-1 was 103.26±59.57, 333.36±42.03, and 827.29±157.11, respectively; and of SW1990 was 245.99±43.97, 494.21±90.01, and 638.76±156.05, respectively (Figure 1). These preliminary experiments showed that the Asensor could successfully infect pancreatic cells effectively.

### Real-time miRNA Function Sorted by Cell Line

Eight miRNAs (miR-200a, -200b, -21, -96, -146a, -10a, -155, -221), which were reported to be related to various pancreatic diseases, were studied in three PDAC cell lines (BxPC-3, CFPAC-1, SW1990), one pancreatic epithelioid carcinoma (PANC-1), and human pancreatic nestin-expressing cells (hTERT-HPNE). The real-time miRNA activity was monitored at 24, 48, and 72 hours after sensor infection.

**Figure 2:**
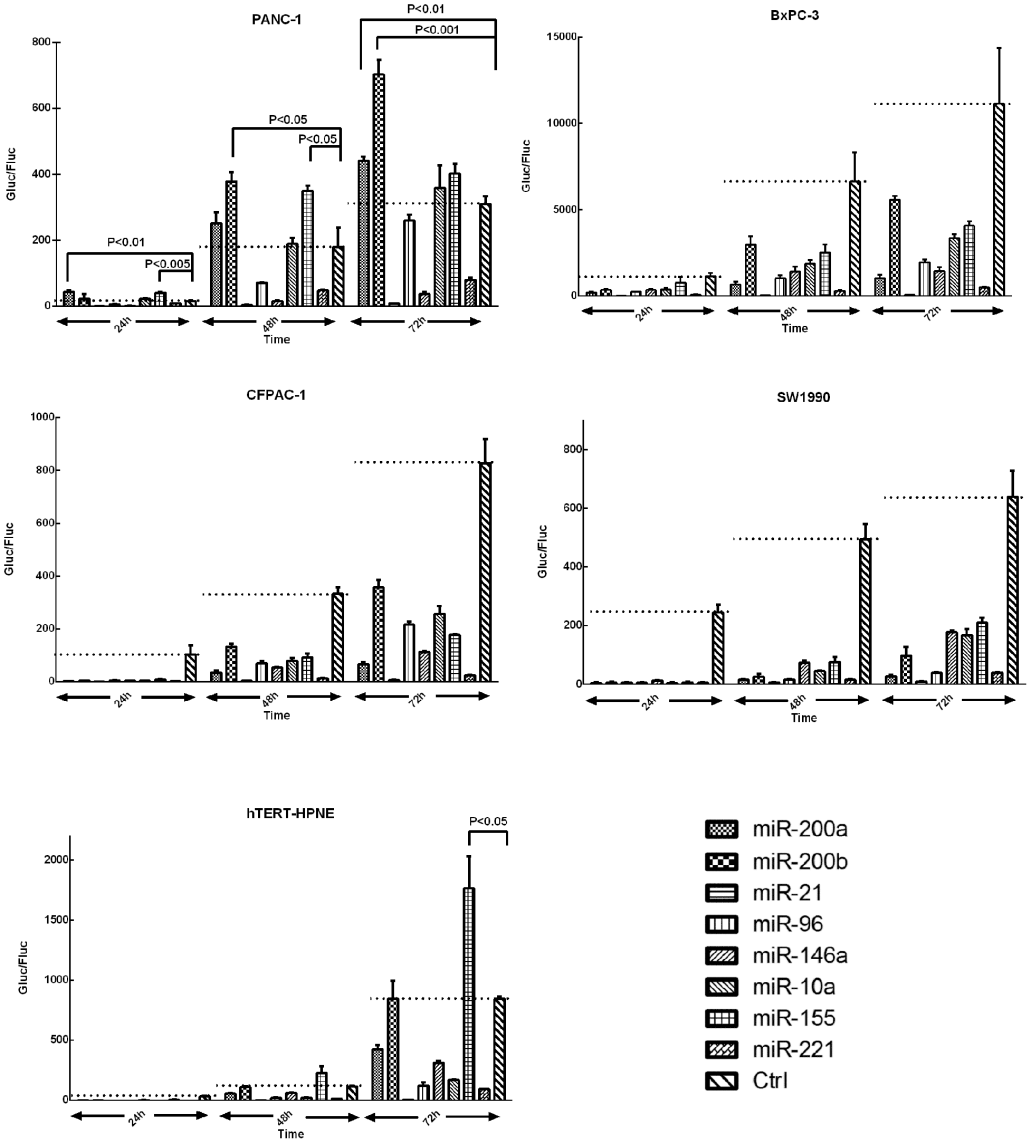
Real-time miRNA function in different cell lines. miRNA activity profiles for PANC-1, BxPC-3, CFPAC-1, SW1990, and hTERT-HPNE were established by using Asensors. miR-200a, -200b, -21, -96, -146a, -10a, -155, and -221 were evaluated at 24, 48, and 72 hours after Asensor infection. To show the overexpression of the report gene (miR-200a, miR-200b, miR-155 in PANC-1 and miR-155 in hTERT-HPNE) compared with control, miRNA activity was expressed as Gluc/Fluc. Data are shown as mean ± SD of triplicate independent experiments. And miRNAs which might upregulated target gene were marked P values.

Like the Asensor control, the Gluc/Fluc of all Asensors were weak within the first 24 hours, but increased greatly within 48 hours (Figure 2). Because the original Gluc and Fluc indices varied among the different cell lines, the ratios of Gluc/Fluc were different; nevertheless, this issue does not hinder the observation of miRNA function.

In each of the three PDAC cell lines, BxPC-3, CFPAC-1, and SW1990, Gluc/Fluc of the eight miRNA Asensors were significantly lower than that of the Asensor control, suggesting that all eight miRNAs displayed a negative control tendency on target protein expression at each time point, which is consistent with current reports and opinion (Figure 2).

Notably, in the PANC-1 cells at 24 hours, the Gluc/Fluc of miR-200a, -200b, -21, -96, -146a, -10a, -155, and -221 was 43.51±8.57, 23.25±24.44, 0.62±0.38, 5.25±2.25, 1.56±0.40, 22.64±2.03, 40.88±5.13, and 9.07±1.47, respectively. Moreover, the Gluc/Fluc of miRNA200a and -155 was significantly higher than that of the control (14.86±5.59, p<0.05), which suggests that these miRNAs might upregulate the expression of the target protein. At 48 hours, the Gluc/Fluc of miR-200a, -200b, -21, -96, -146a, -10a, -155, and -221 was 251.81±58.32, 377.90±51.11, 3.56±1.42, 71.65±1.17, 15.46±4.98, 189.34±31.41, 350.06±27.48, and 48.72±3.66, respectively. The Gluc/Fluc of the control at 48 hours was 178.98±102.26 and was significantly lower than that of miR-200b and -155 (p = 0.039 and 0.049, respectively). At 72 hours, the Gluc/Fluc of miR-200a, -200b, -21, -96, -146a, -10a, -155, and -221 was 441.17±22.52, 702.38±76.97, 8.43±2.74, 260.46±29.95, 36.82±13.61, 358.30±118.84, 403.13±49.49, and 79.98±12.87, respectively. The Gluc/Fluc of the control at this time point was 309.71±41.42, which was significantly lower than that of miR-200a and -200b (p = 0.008 and 0.001, respectively) (Figure 2). In brief, in PANC-1 cells, some miRNAs displayed upregulation of target protein expression, and miRNA function varied greatly among the tissue types and time courses, suggesting that the pancreatic epithelioid carcinoma cell line was different from PDAC cell lines in miRNA relative pathophysiology.

In hTERT-HPNE cells, the Gluc/Fluc of miR-200a, -200b, -21, -96, -146a, -10a, -155, -221, and the controls at 24 hours was 2.02±0.78, 2.77±0.21, 0.19±0.04, 0.65±0.14, 2.69±0.66, 0.58±0.25, 6.81±0.94, 0.58±0.05, and 31.85±9.72, respectively; at 48 hours, 55.95±12.37, 110.56±12.14, 1.45±0.10, 19.20±9.35, 61.73±3.93, 21.29±5.47, 226.71±96.15, 12.26±4.30, 115.53±14.20, respectively; and at 72 hours, 422.10±63.60, 846.77±257.91, 6.20±0.68, 122.82±48.36, 312.92±22.99, 168.25±13.17, 1768.22±458.70, 92.34±4.47, and 845.77±31.66, respectively. The miRNA profile in hTERT-HPNE cells was obviously different from the PANC-1 and PDAC cell lines. At 72 hours, the Gluc/Fluc of miR-155 was significantly higher than that of the control (p = 0.025), which suggested again that some miRNAs could upregulate the target protein expression (Figure 2).

### Real-time miRNA Function Sorted by miRNAs

**Figure 3:**
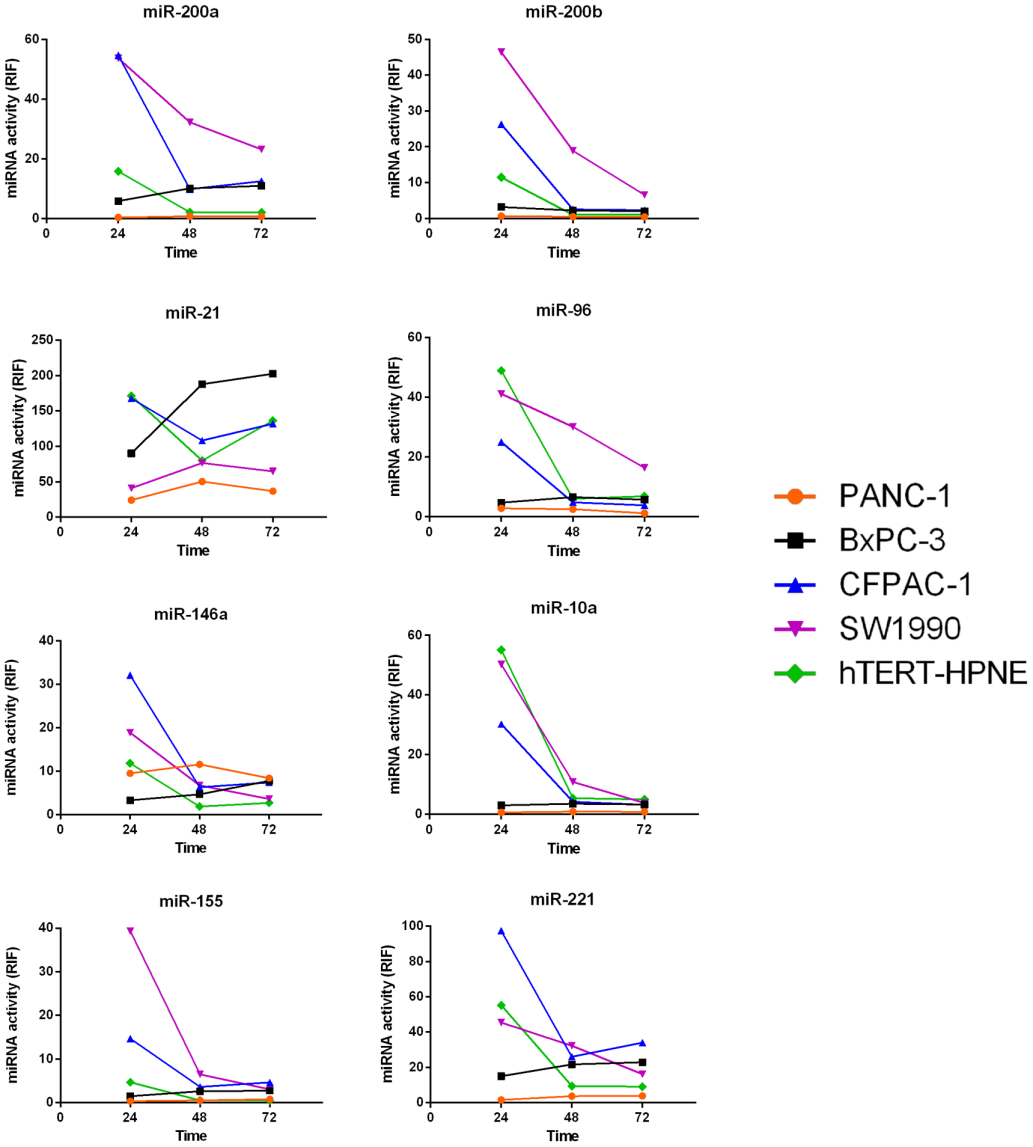
Line chart of every miRNA in different cell lines at 24, 48, and 72 hours. CFPAC-1 and SW1990, metastatic carcinomas, showed high RIF at 24 hours, which dropped rapidly from 24 to 48 hours. miRNAs of PANC-1 consistently showed low activities in almost all cell lines as well as upregulation of the target protein. However, all RIFs in miR-21 were high and stable.

All eight miRNAs displayed different features in different cell lines, as described above. When the data were classified by each miRNA and represented by RIF, much new information came out. Almost all miRNAs displayed the highest activity within the first 24 hours, and their activity decreased with time, except for miR-21. The miRNA activity of miR-21 was about three times higher than that of the others in all cell lines and was almost stable with time. In PANC-1 cells, miR-200a, 200b, -96, -10a, -155, and -221 maintained the lowest activity at all time points (Figures 2 and 3). In SW1990 cells, miR-200a, -200b and -96 maintained the highest activity within 72 hours (Figure 3). Taken together, miRNA activity was related to cell/tissue types and time course, and some miRNAs could upregulate gene expression.

## Discussion

With the identification of a vast number of miRNAs whereby each one carries a long list of putative targets, the challenge is now to understand their biological function, and Asensors provide a method for the further understanding of miRNAs. Because target gene expression and cell signaling are not independent among miRNAs, studying them in organisms is considered more objective than using lysed cells.

To investigate real-time miRNA function in pancreatic cancer, we selected four cell lines derived from pancreatic cancer: PANC-1 (pancreatic epithelioid carcinoma), BxPC-3 (pancreatic adenocarcinoma), CFPAC-1 (liver metastasis of pancreatic ductal adenocarcinoma; cystic fibrosis), and SW1990 (spleen metastasis of a grade II pancreatic adenocarcinoma). hTERT-HPNE, a no-cancer cell line, was selected as the normal pancreas control. Although the miRNA profiles were diverse, based on our data, these five cell lines could be divided into three groups. The adenocarcinoma cells, BxPC-3, CFPAC-1, and SW1990, were similar in eight miRNA profiles, all miRNAs negatively regulated expression of the target mRNA, and they shared a similar tendency of miRNA activity. The epithelioid carcinoma PANC-1 cell line was significantly different from the other four cell lines, with many of the miRNAs displaying upregulation of the target mRNA expression, which deviates from the current opinion on miRNA function. Our data indicated that miRNA profiles were vastly different among all cell lines, and the miRNA profile showed a possible co-relationship with the pathophysiology of pancreatic cancer.

miRNA function is dynamic and varied throughout the time course; thus, current methods such as Northern blot, RT-PCR, and microarrays cannot accurately reveal the real-time dynamic function of any miRNA. For example, using Northern blot analysis, pri-miRNA, pre-miRNA, and mature miRNA can be distinguished, but the sensitivity of the assay is relatively low. Stem-loop RT-PCR can detect the copy number of mature miRNAs with high sensitivity, but specific primers are required, and it is difficult to perform in a high-throughput manner. In our study, we used a new method called “Asensor” to monitor the functions of miRNA in live cells. Although RT-PCR was used quantify miRNA, the cells had to be lysed, which prevented the acquisition of real-time and dynamic results. Thus they were not comparable. Microarrays are suitable for the high-throughput detection of many miRNAs, but it cannot distinguish between pri-miRNA, pre-miRNA, and mature miRNA, and the results are often not reproducible due to variations in miRNA quality. Importantly, the results from these methods only represent quantitative results and cannot reflect miRNA activity that directly involves a post-transcriptional regulation of gene expression. Therefore, our study provided a new method to observe miRNA activity in molecular biology research. In addition, Gluc possesses a natural secretory signal and, upon expression, is secreted into the cell medium. The Gluc-containing samples can be stored at −20°C for long-term storage or at 4°C for several days without loss of activity. Therefore, cell lysis is not necessary, and it is convenient to monitor the real-time function of miRNA.

Moreover, through dynamic observation, the function of miRNAs was different among these cell lines. There are different subsets in the biological characteristics of PDAC, and miRNAs play a different role in different subsets. For example, as previously shown, the Gluc level was significantly higher in BxPC-3 cells, since BxPC-3 is a poorly differentiated human pancreatic cancer cell line with hypermetabolism, which suggests that the expression of Gluc and Fluc may depend on the metabolism of the cells. In addition, PANC-1 and hTERT-HPNE were similar in that microscopic examination of PANC-1 showed it to be an undifferentiated carcinoma, but ducts lined with markedly dysplastic or frankly malignant-type cells were observed in certain regions [14]. hTERT-HPNE was originally isolated from the ductal structure of a human pancreas and immortalized by expression of the catalytic subunit of telomerase (hTERT) [15]. hTERT can immortalize primary human cells without changing their phenotypic properties or causing cancer-associated changes [16]–[19]. Mounting evidence now suggests that acinar-to-ductal metaplasia plays a vital role in the initiation of pancreatic cancer development [20]–[23]. hTERT-HPNE cells have properties similar to that of the intermediary cells produced during acinar-to-ductal metaplasia. The properties shared by hTERT-HPNE and these intermediary cells included their undifferentiated phenotype and the ability to differentiate into pancreatic ductal cells [24]. It seems that PANC-1 and hTERT-HPNE both possess the characteristic of being intermediary or undifferentiated cells. Thus, some target genes up-regulated by miRNA in the two cell lines may participate in tumorigenesis.

It is often reported that miRNAs negatively regulate post-transcriptional gene expression by inhibiting translation and causing degradation of the target mRNA [13], primarily through base-pairing interactions, which leads to either mRNA degradation or translational inhibition, depending upon the degree of match between the “seed sequence” (positions 2–7 at the 5′ side) of the miRNA and 3′-UTR of the mRNA. When the seed sequence perfectly or partially matches with target 3′-UTR of the mRNA, then it may lead to degradation of the mRNA or inhibit translation [25]–[28]. The expression profiles of miRNAs are frequently altered in tumors, and in some cases, a reduction in the expression of miRNA may cause increased expression of the oncogenic target genes [29]. The biological functions of miRNAs are highly dependent on cellular context, which may be due to the differential expression of their target mRNAs. It has also been demonstrated that cellular proteins can also regulate RNAi. Therefore, according to the results of our experiments using dynamic monitoring of miRNA function, the classic theory that miRNAs negatively regulate gene expression by inhibiting translation and causing degradation of the target mRNA is not completely correct. The characteristic of high heterogeneity demonstrates that the abundance of miRNA is influenced by various factors such as different cell origins, cellular metabolism, antigen expression, cellular productions, and so on. When miRNA abundance is relatively low, it may activate other signal transduction pathways or induce some factors promoting target gene upregulation, eventually leading to the overexpression of target genes. On the other hand, AU-rich elements (AREs) and miRNA target sites are conserved sequences in mRNA 3′UTRs that control gene expression posttranscriptionally. In 2007, Vasudevan et al found that the TNFα ARE recruits miR369-3 to mediate translation up-regulation in serum-starved conditions and to cause repression in synchronized proliferating cells [30]. miRNAs oscillate between repression and activation in coordination with the cell cycle: In proliferating cells they repress translation, whereas in G1/G0 arrest (which often precedes differentiation), they mediate activation. This regulation occurs on at least two levels. First, recruitment of the microRNP reflects both its expression level and its ability to productively interact with mRNA target sites. Second, the AGO2 complex must be subject to modification because tethered AGO2 differentially regulates translation according to cell growth conditions. Thus, based on our findings, miR-200a, miR-200b, and miR-155 induced the overexpression of the reporter gene in PANC-1, and miR-155 induced overexpression of the gene in hTERT-HPNE. These two cell lines are similar in that they are undifferentiated or had intermediary cells that included their undifferentiated phenotype. Thus, we inferred that miRNA might up-regulate target gene expression and may play an important role prior to differentiation.

As we know, PDAC is a heterogeneous disease. Comprehensive genetic analysis has shown that pancreatic cancers contain an average of 63 exomic alterations in 12 key cellular signaling pathways, although not every pathway is altered in every pancreatic tumor [31]–[33]. This suggests that different pancreatic cell lines, even different PDAC patients, can have altered cellular signaling pathways involved in tumorigenesis and development. Our conclusion that miRNAs are regulatory factors of biological processes that can be regulated themselves is apprehensible.

All of these miRNAs had their own target gene and mechanism (Table 1). As previously shown, miR-21 obviously inhibited Gluc expression in all cell lines. It was previously reported that miR-21 is amplified in many malignant diseases, which supports our results. However, its specificity was unsatisfactory; miR-21 was reportedly increased in many types of cancer as well as the no-cancer cell line hTERT-HPNE in our study. Other miRNAs do not have consistent results in every PDAC cell line, although they might be aberrant in PDAC compared with no-cancer cell lines, as reported previously. Distinguishing these different molecular partings of the therapeutic targets will help improve individual PDAC treatment and overall prognosis of the disease.

**Table 1:** The expression, biological function, and mechanism of analyzed candidate microRNAs.

microRNA	Tumor	Blood	Pancreatic fluid	Predicted biological function	Gene targets or mechanism	Refs
miR-200a,b	↑	↑		oncogene	ZEB1, ZEB2, EMT	[34]–[37]
miR-21	↑	↑	↑	oncogene	PTEN, RECK, PDCD4,TPM1	[38]–[41]
miR-96	↓			tumor suppression	K-RAS	[42]
miR-146a	↓			tumor suppression	EGFR, MTA-2, IRAK-1, NF-kB	[43]
miR-10a	↑			oncogene	HOXB1, HOXB3, cadherin/catenin, E-cadherin	[44]
miR-155	↑	↑	↑	oncogene	TP53INP1, ROS, HIF1α activity	[40], [45]–[46]
miR-221	↑			oncogene	CDKN1B	[41], [47]

ZEB: zinc finger E-box binding homeobox. EMT: epithelial-mesenchymal transition. PTEN: phosphatase and tensin homolog. RECK: reversion-inducing-cysteine-rich protein with kazal motifs. PDCD4: programmed cell death 4. TPM1: tropomyosin 1. EGFR: epidermal growth factor receptor. MTA-2: metastasis associated 1 family, member 2. IRAK-1: interleukin-1 receptor-associated kinase 1. NF-kB: nuclear factor of kappa B. HOXB: homeobox B cluster. TP53INP1: tumor protein p53 inducible nuclear protein 1. ROS: reactive oxygen species. HIF1α: hypoxia inducible factor 1, alpha subunit. CDKN1B: cyclin-dependent kinase inhibitor 1B.

In conclusion, we present a pilot study demonstrating the feasibility of miRNA analysis using Asensors as a high-throughput real-time consecutive functional method as an alternative to current popular methods, which do not accurately show real-time miRNA function. It was confirmed that certain miRNAs could upregulate gene expression in PANC-1 and hTERT-HPNE cells, suggesting that miRNA might participate in the pathophysiology of pancreatic cancer. Thus, this report provides a convenient, accurate, and sensitive approach to miRNA research. Future tissue profiling studies will be continued to help optimize miRNA functional studies in patients with malignant and benign pancreatic diseases, which are much different from *in vitro* studies.
